# Ocular involvement in Behçet’s disease: relevance of new diagnostic tools

**DOI:** 10.1093/rap/rkaa038

**Published:** 2020-08-10

**Authors:** Maria Giulia Tinti, Filippo Vaira, Michele Inglese, Gaetano Serviddio, Salvatore De Cosmo, Daniela Marotto, Angelo De Cata

**Affiliations:** r1 Unit of Internal Medicine, ‘Casa Sollievo della Sofferenza’ Hospital, IRCCS, San Giovanni Rotondo; r2 Department of Medical and Surgical Sciences, University of Foggia, Foggia; r3 Vaira Ophthalmology Clinic, Manfredonia; r4 Rheumatology Unit, ATS Sardegna, ‘P. Dettori’ Hospital, Tempio Pausania, Italy

**Key message**

- Spectral domain optical coherence tomography and optical coherence tomography angiography can be valuable for recognizing early retinal ischaemic changes in patients with Behçet’s disease.

Sir, Ocular involvement in Behçet’s disease (BD) is characterized by a relapsing non-granulomatous uveitis that can affect both the anterior and the posterior segment of the eye, often causing a decrease of visual acuity that can progress to blindness [1]. This takes place particularly when the recurrent inflammatory flares of the disease translate to damage of the posterior eye segment, leading to a permanent injury of the retinal vasculature [1]. The early identification of disease activity is still challenging, although its prompt recognition can decrease the risk of poor visual outcome [2]. Spectral domain optical coherence tomography (SD-OCT) and optical coherence tomography angiography (OCTA) could complement the nformation provided by the standard fluorescein angiography (FA).

Herein, we report the case of a 39-year-old Caucasian man with BD, who came to our department for his annual evaluation. The patient gave written informed consent for this case report. Seven years ago, he was diagnosed with BD based on the recurrence of oral and genital ulcers, skin lesions and a positive pathergy test. He also had an anterior right uveitis and an asymmetrical polyarthritis, currently deserving only a low-dose of methylprednisolone (4 mg/day). His medical history was otherwise unremarkable.

**Fig. 1:**
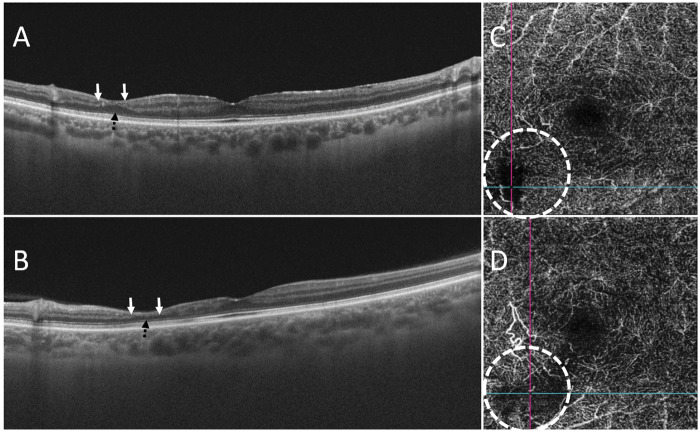
Spectral domain optical coherence tomography and optical coherence tomography angiographic features of paracentral acute middle maculopathy Spectral domain optical coherence tomography scan of the right (**A**) and left (**B**) eye reveals thinning of the inner nuclear layer (top arrows) and irregularity of the outer plexiform layer (bottom interrupted arrow). Optical coherence tomography angiography scan shows the corresponding area of non-perfused deep capillary plexus (dashed circle) in the right (**C**) and left (**D**) eye.

At current presentation, he reported that two episodes of transient bilateral scotomas had occurred 3 months before. His best-corrected visual acuity was 20/20 in the right eye (OD) and 20/25 in the left eye (OS). Both fundus examination and FA were normal. However, SD-OCT revealed a bilateral limited area of severe thinning of the retinal inner nuclear layer, indicating a partial collapse of the overlying retinal structures (Fig. 1A and B). Complementary information was provided by OCTA, which showed bilaterally an isolated ischaemia of the deep capillary plexus corresponding to the atrophic lesions detected by SD-OCT (Fig. 1C and D). These findings, documenting subtle ischaemic changes, were consistent with the chronic evolution of a paracentral acute middle maculopathy. Therefore, AZA 100 mg/day (1.5 mg/kg/day) was added to the therapy, and we planned a tighter ocular follow-up aimed to identify further ocular disease progression.

The retinal capillary network is a multilayered structure, composed of the superficial, intermediate and deep capillary plexuses [3]. FA, the current reference method for the assessment of posterior ocular involvement in BD, allows only a two-dimensional study of the superficial retinal circulation, and frequently, the damage to retinal vessels limits the reliable assessment of disease activity [3]. SDOCT better defines the morphology of the retina, whereas OCTA can evaluate its superficial and deep circulation separately, simulating angiographic images [2, 4]. Through these techniques, we identified the ocular involvement of our patient, whose deep retinal capillary plexus was severely affected. Indeed, paracentral acute middle maculopathy, whose clinical course might be asymptomatic or characterized by transient or persistent paracentral scotomas, is caused by a focal ischaemia of the deep capillary plexus [5]. This disorder can be detected in eyes with compromised retinal arterial circulation in the absence of FA abnormalities as an isolated phenomenon or as the expression of an underlying systemic disease, as occurred in our patient [4, 5]. The posterior eye involvement is a prognostic factor of poor visual prognosis, especially in young men. In our patient, we added a dose of AZA apt to allow a better control of the disease, because our findings were the expression of a past ocular flare, probably responsible for the scotomas. In the subsequent 6 months, the patient did not experience further episodes of scotomas.

SD-OCT and OCTA, by yielding an in-depth investigation of retinal vascular pathologies, could allow us to identify unrecognized ocular BD cases. In fact, our SD-OCT and OCTA findings are in line with those of a recent study reporting that patients with a diagnosis of BD and without significant FA perfusion abnormalities did have significant damage of the deep capillary plexus [6]. It is conceivable that the ischaemia of the deep capillary plexus, situated in a watershed region of oxygen supply, might be an early expression of BD. This could also account for the progressive loss of vision observed in some patients with BD and negative FA findings and could possibly be useful screening tools for this condition [7]. Therefore, methods appropriate for exploring the retinal circulation more accurately might provide evidence for earlier stages of the disease.

Overall, the findings we obtained with SD-OCT and OCTA would suggest that the prevalence of ocular involvement might be higher than expected in BD. This might have implications for treatment, such as a lower threshold for starting a therapy. In summary, the present case outlines that SD-OCT and OCTA can be of value in the evaluation and monitoring of BD patients. These techniques might provide a more comprehensive assessment of retinal inflammatory involvement, revealing subtle ischaemic changes that precede clinically overt ocular BD.

*Funding*: No specific funding was received from any bodies in the public, commercial or not-for-profit sectors to carry out the work described in this article.

*Disclosure statement*: The authors have declared no conflicts of interest.
